# High-throughput metabolic screening of microalgae genetic variation in response to nutrient limitation

**DOI:** 10.1007/s11306-015-0878-4

**Published:** 2015-11-13

**Authors:** Amit K. Bajhaiya, Andrew P. Dean, Thomas Driver, Drupad K. Trivedi, Nicholas J. W. Rattray, J. William Allwood, Royston Goodacre, Jon K. Pittman

**Affiliations:** Faculty of Life Sciences, The University of Manchester, Michael Smith Building, Oxford Road, Manchester, M13 9PT UK; Department of Geography, University of Sheffield, Sheffield, S10 2TN UK; School of Chemistry, Manchester Institute of Biotechnology, The University of Manchester, 131 Princess Street, Manchester, M1 7DN UK; Environmental & Biochemical Sciences Group, The James Hutton Institute, Invergowrie, Dundee, DD2 5DA Scotland, UK

**Keywords:** Metabolite screening, FT-IR spectroscopy, Microalgae, Lipids, Starch, *Chlamydomonas reinhardtii*

## Abstract

Microalgae produce metabolites that could be useful for applications in food, biofuel or fine chemical production. The identification and development of suitable strains require analytical methods that are accurate and allow rapid screening of strains or cultivation conditions. We demonstrate the use of Fourier transform infrared (FT-IR) spectroscopy to screen mutant strains of *Chlamydomonas reinhardtii*. These mutants have knockdowns for one or more nutrient starvation response genes, namely *PSR1*, *SNRK2.1* and *SNRK2.2*. Limitation of nutrients including nitrogen and phosphorus can induce metabolic changes in microalgae, including the accumulation of glycerolipids and starch. By performing multivariate statistical analysis of FT-IR spectra, metabolic variation between different nutrient limitation and non-stressed conditions could be differentiated. A number of mutant strains with similar genetic backgrounds could be distinguished from wild type when grown under specific nutrient limited and replete conditions, demonstrating the sensitivity of FT-IR spectroscopy to detect specific genetic traits. Changes in lipid and carbohydrate between strains and specific nutrient stress treatments were validated by other analytical methods, including liquid chromatography–mass spectrometry for lipidomics. These results demonstrate that the *PSR1* gene is an important determinant of lipid and starch accumulation in response to phosphorus starvation but not nitrogen starvation. However, the *SNRK2.1* and *SNRK2.2* genes are not as important for determining the metabolic response to either nutrient stress. We conclude that FT-IR spectroscopy and chemometric approaches provide a robust method for microalgae screening.

**Electronic supplementary material:**

The online version of this article (doi:10.1007/s11306-015-0878-4) contains supplementary material, which is available to authorized users.

## Introduction

Algae have long been recognized as a promising resource for biotechnological applications, such as a source of nutritional supplements like omega-3 fatty acids or carotenoids, or as a feedstock for biofuel generation (Driver et al. 2014; Guccione et al. 2014; Guedes et al. 2011; Larkum et al. 2012). For example, some species of unicellular microalgae are able to synthesise large amounts of neutral lipids (up to 60 % of cell dry weight in many cases), which are stored as triacylglycerol (TAG) and can be easily converted into fatty acid methyl esters to be used as biodiesel (Chisti 2008; Hu et al. 2008). In contrast, some species of microalgae are good sources of carbohydrates, particularly by producing high quantities of starch, which could be used as a fermentation feedstock or industrial product (Branyikova et al. 2011). There is a wide natural diversity of algae, with over 70,000 species determined from some recent estimates (Guiry 2012), plus further potential diversity that can be generated by mutagenesis (Cagnon et al. 2013); together this provides a large resource of distinct chemicals and metabolites that could be screened and identified. However, this tremendous diversity provides the challenge of how to screen efficiently and rapidly naturally occurring and genetically modified algae in order to provide accurate identification and quantification of these chemicals and metabolites.

Several conventional methods of metabolite quantification including thin layer chromatography, high-performance liquid chromatography (HPLC), and gas chromatography (GC), often coupled with mass spectrometry (MS), are not particularly amenable for high-throughput screening due to high cost and the requirement for large amounts of biomass, as well as the need for cell extraction and preparation, which can be time-consuming and technically laborious. Fluorescent metabolite reporters, like the neutral lipid stains Nile Red or Bodipy, can allow reasonably fast and high-throughput screening, particularly when coupled to flow cytometry methods (Elliott et al. 2012; Manandhar-Shrestha and Hildebrand 2013), but these dyes are unfortunately limited to the detection of a single metabolite at a time and there can be challenges due to variation in efficiency of dye accumulation or photo-bleaching (Dean et al. 2010). By contrast, there are alternative analytical methods that are more suitable for metabolomic screening of strain collections. One of these is Fourier transform infrared (FT-IR) spectroscopy, which is a rapid, high-throughput and non-destructive analytical method that provides a robust metabolic fingerprint of a sample (Ellis et al. 2012). FT-IR spectroscopy can reliably assess the macromolecular composition of algae and provide an accurate quantification of lipid and starch accumulation (Dean et al. 2010; Laurens and Wolfrum 2011; Meng et al. 2014; Stehfest et al. 2005). Furthermore, when coupled with microscopy it can be used to generate chemical maps of algae to show the cellular distribution of lipid and starch storage (Patel et al. 2008). The complex spectral data can be analysed using multivariate statistical tools to identify metabolic characteristics that are diagnostic markers for a particular trait (Dean et al. 2010; Ellis et al. 2002; Nicolaou and Goodacre 2008) such as oil production, starch production, or other primary or secondary metabolites.

FT-IR spectroscopy has been used previously to detect metabolic changes within individual algae, such as in response to nitrogen (N) or phosphorus (P) limitation (Dean et al. 2008, 2010; Stehfest et al. 2005), which are reliable inducers of storage carbon metabolite biosynthesis, in particular TAG and starch (Driver et al. 2014; Merchant et al. 2012). Furthermore, it has been suggested that FT-IR spectroscopy can be used to differentiate between microalgal species (Bounphanmy et al. 2010; Driver et al. 2015). However, to our knowledge, this metabolic fingerprinting method has not yet been validated as a tool for screening microalgal mutants or to discriminate between mutant lines. In this study, we have demonstrated FT-IR spectroscopy screening of 10 *Chlamydomonas reinhardtii* strains, including a wild type and nine mutant strains, grown under N and P limitation conditions. Three sets of mutants were chosen that are mutated for one or more of the *PSR1*, *SNRK2.1* or *SNRK2.2* genes, which are known to control responses to starvation of different nutrients. *PSR1* (*P starvation response 1*) encodes a transcription factor that regulates the response to P starvation (Wykoff et al. 1999). *SNRK2.1* and *SNRK2.2* (*SNF1*-*related protein kinase 2.1* and *2.2*) encode serine/threonine kinases and are related to SNRK proteins from higher plants and yeast which are known to regulate aspects of carbon metabolism (Ghillebert et al. 2011). In addition, *SNRK2.1* and *SNRK2.2* show some genetic interaction with *PSR1* in response to P starvation (Moseley et al. 2009), but none of these genes are known to be required for the response to N starvation. Furthermore, we have recently shown that the *psr1* mutant has impaired lipid and starch biosynthesis under P starvation conditions, demonstrating a role of PSR1 in controlling P starvation-specific metabolite regulation (Bajhaiya et al. unpublished). Thus the *psr1* mutant is a good control strain for which to test the sensitivity of FT-IR spectroscopic analysis. We demonstrate here that multivariate analysis of FT-IR spectra can clearly discriminate between the tested wild type and mutant lines of *C. reinhardtii* and in particular distinguish all lines with a *psr1* mutant background. Moreover, we find that *snrk2.1* and *snrk2.2* mutations do not cause significant metabolic changes under any of the cultivation conditions.

## Materials and methods

### Strains, culture conditions and physiological analysis

Wild type *C. reinhardtii* (CC125) and nine previously generated mutant lines comprising of single, double or triple mutants of the *PSR1*, *SNRK2.1* and *SNRK2.2* genes were used (Supplementary Table 1). All strains were grown photo-heterotrophically as sterile axenic batch cultures in standard Tris–acetate-phosphate (TAP) medium containing 1 mM P (as K_2_HPO_4_/KH_2_PO_4_) and 7 mM N (as NH_4_Cl), and in modified TAP media containing different concentrations of N (0.07, 0.3, 0.7, or 3.5), but with P held constant at 1 mM, and different concentrations of P (1 μM, 0.01, 0.05, or 0.1), but with N held constant at 7 mM, essentially as described previously (Webster et al. 2011). When the concentration of N or P was reduced, the concentrations of all other components in TAP medium were also kept constant. Growth of all strains over time was measured by optical density measurements at 680 nm (OD_680nm_) using a Jenway UV–Visible spectrophotometer. Fresh-weight biomass of the culture samples was determined by centrifugation at 1500×*g* for 20 min in a pre-weighed tube. Total chlorophyll (chlorophyll *a* and *b*) measurement of day seven cells was performed by harvesting 5 mL of cells by centrifugation (3000×*g* for 10 min), resuspending the pellet in 80 % acetone and vortexing to extract the pigments with cellular debris removed by centrifugation. The concentration of chlorophyll *a* and *b* was determined by measuring absorbance of the extract as described previously (Porra et al. 1989). In vivo chlorophyll fluorescence was measured using a pulse-modulated fluorometer (PAM Walz 101) by taking 1 mL of day seven culture in a suspension cuvette and Aquire 3.2 software was used to calculate the ratio of variable fluorescence *F*_v_ to maximal fluorescence *F*_m_ (*F*_v_/*F*_m_) (Maxwell and Johnson 2000).

### FT-IR spectroscopy

A 1 mL of sample from each replicate culture was added to a pre-weighed Eppendorf tube and centrifuged at 14,000×*g* for 5 min at room temperature and the supernatant was removed. The biomass was weighed and normalised to 60 mg mL^−1^ by addition of Milli-Q (Millipore) water then 30 µL of normalized biomass was deposited onto a 96-well silicon microplate and oven-dried at 40 °C overnight. The first well of each plate was left blank for background measurement. The plate was placed in a HTS-XT high-throughput microplate extension and spectra were collected using a FT-IR spectrometer (Bruker Equinox 55 FT-IR spectrometer), equipped with a deuterated triglycerine sulfate (DTGS) detector. The absorbance spectra were measured over the wavenumber range 4000–600 cm^−1^. Generated data were imported into MATLAB v. 2010a (The MathWorks) and spectra were baseline corrected using extended multiplicative scatter correction (EMSC) (Martens and Stark 1991). The band heights for total lipid (1740 cm^−1^), amide I (1655 cm^−1^) and carbohydrate (1160, 1086, 1050 and 1036 cm^−1^) (see Supplementary Fig. 1) were measured individually and lipid:amide I and carbohydrate:amide I ratios of these band heights were calculated.

### Lipid measurement

For ultra high performance liquid chromatography–mass spectrometry (UHPLC–MS) analysis of lipids, 30 mg fresh weight of Milli-Q water-washed algal material was snap frozen in liquid nitrogen and ground using a Retsche ball mill with 2 mm stainless steel ball bearings then 1 mL of methanol:chloroform:water (2.5:1:1) was added. Samples were shaken at room temperature for 15 min and centrifuged at 14,000×*g* for 10 min. 1 mL of supernatant was removed, 0.5 mL of water added and mixed thoroughly then centrifuged for 10 min. The non-polar lower chloroform phase was removed and dried at 40 °C for 1–2 h until completely dry. UHPLC–MS analysis was carried out on an Accela UHPLC autosampler system coupled to an electrospray LTQ-Orbitrap XL hybrid mass spectrometry system (ThermoFisher, Bremen, Germany). Analysis was carried out in positive ESI mode whilst each run was completely randomised to negate for any bias. A water/methanol gradient type UHPLC method was used during each run as is previously described (Allwood et al. 2015; Wedge et al. 2011). 10 µL of the extract was injected onto a Hypersil GOLD UHPLC C_18_ column (length 100 mm, internal diameter 2.1 mm, particle size 1.9 µm, Thermo-Fisher Ltd. Hemel Hempsted, UK) held at a constant temperature of 50 °C whilst a solvent flow rate of 400 µL min^−1^ was used to drive the chromatographic separation. Data processing was initiated by the conversion of the standard MS raw files to the universal NetCDF format using Xcalibur software (Thermo-Fisher Ltd. Hemel Hempsted, UK). In house peak deconvolution software containing the XCMS algorithm (http://masspec.scripps.edu/xcms/xcms.php) was used for pick picking (Dunn et al. 2008) generating a data matrix of mass spectral features with related accurate *m*/*z*, retention time pairs and peak areas. Data from the internally pooled QC samples were used to align for instrument drift and quality control via an in house MATLAB LOESS alignment script. The data matrix was signal corrected to remove peaks that crossed the 20 % RSD threshold within QC samples across the analytical run. Data was normalised on the basis of fresh weight biomass. Identification of lipid features was performed applying the PUTMEDID-LCMS set of workflows as previously described (Brown et al. 2011). Neutral lipids were also measured by Nile Red fluorescence staining as described previously (Chen et al. 2009; Dean et al. 2010). Day seven cell samples were normalised to an OD_680nm_ value of 0.5 by dilution then 10 µL of Nile Red (50 µg mL^−1^) was added to a 1 mL final volume of diluted cells and incubated for 10 min at room temperature. Fluorescence of Nile Red-stained neutral lipid was measured using a Hitachi F-2000 fluorescence spectrometer (excitation 530 nm/emission 575 nm). The concentration of neutral lipids was determined using a triolein standard.

### Starch and protein measurement

Total protein was quantified by the Bradford assay. A 2 mL sample of day-seven culture was centrifuged at 1500×*g* for 10 min then resuspended in extraction buffer (30 mM Tris HCl, pH 7.5, and 1 µL of protease inhibitor cocktail) followed by two rounds of freezing/thawing in liquid N_2_. The extract was centrifuged at 12,000×*g* for 15 min and protein determination of the supernatant was performed using the Bradford dye assay kit (Bio-Rad) using bovine serum albumin (BSA) as a standard. For starch determination, a 5 mL sample of day-seven culture was centrifuged at 1500×*g* for 10 min and the pellet was washed with 500 µL of 80 % ethanol to remove chlorophyll. Cells were incubated at 85 °C for 5 min and centrifuged at 13,000×*g* for 10 min. Pellets were resuspended in 200 µL of 80 % ethanol, 500 µL DMSO and incubated at 90 °C for 1 h in a Thermo shaker to break cells and solubilize the starch. Total starch was quantified using a Total Starch Assay kit (Megazyme) using the manufacturer’s specifications. Starch concentration was determined using a d-glucose standard curve and values were multiplied by 162/180 (adjustment for free d-glucose to anhydro d-glucose) to calculate total starch.

### Multivariate data analysis

Principal component analysis (PCA) and principal component-discriminant function analysis (PC-DFA) was performed using an in-house MATLAB algorithm to identify any differences between the FT-IR spectra, essentially as described previously (Allwood et al. 2006). Further analysis of spectral data was performed by partial least squares-discriminant analysis (PLS-DA) using Unscrambler v.10.1 (CAMO Software AS) in order to model the classification of nutrient stressed and mutant-derived spectra. For the generation of classification models, replicate spectra from non-stressed and stressed strains, and wild type and mutant strains were used as training data sets then independent replicate spectra from all strains were used to validate the models. Data sets were assigned into training and testing sets, using a random number generator. The training set was used to generate the PLS model, and the validation set was used to assess the PLS model’s ability to assign samples from these sets into the correct group. Initially, a PLS regression was performed on the training set using a cut down (1800–950 cm^−1^) spectra. Samples were assigned to specific category variables according to their identity (e.g. high P/N, low N, low P or wild type, *psr1* background, *snrk* background) and these variables were split to generate numerical assignments. These categories were used as predictors in the running of the PLS regression. The PLS regression was performed with a maximum of seven components, constant X and Y weights, and using a full cross-validation. This PLS regression was then used as a model for the PLS-DA predictive analysis of the validation data set with seven components, using full prediction settings. The resulting predictive Y values and 95 % confidence intervals for each predictive measure were plotted. For statistical analysis of physiological and biochemical data, differences between treatments and cell lines were assessed using one-way ANOVA performed using IBM SPSS Statistics version 20. When significant differences were detected at a *P* value <0.01, the Tukey post hoc test was applied.

## Results and discussion

### Discrimination of nutrient limitation responses

**Fig. 1:**
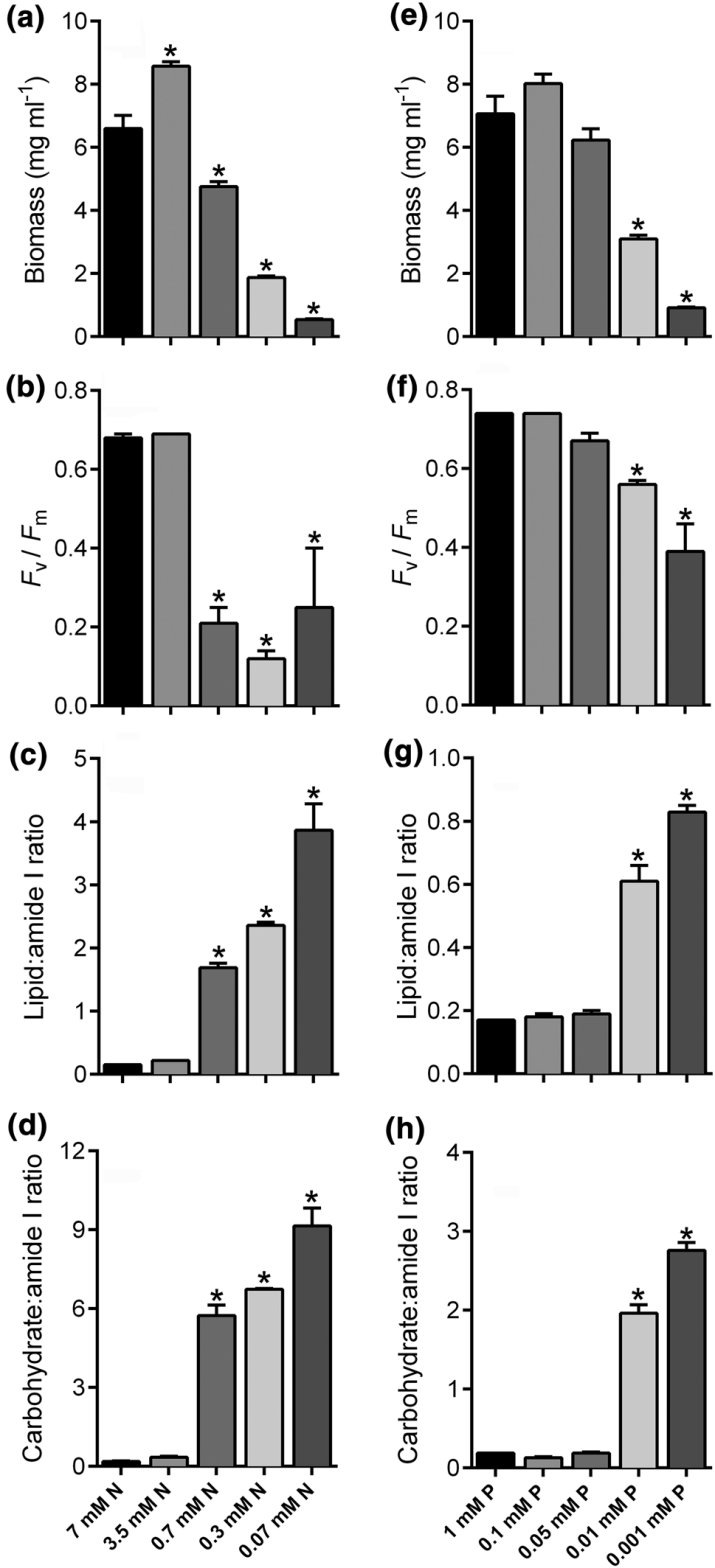
Screening nutrient limitation conditions. Fresh weight biomass (**a**, **e**), chlorophyll fluorescence (*F*_v_/*F*_m_ ratio) (**b**, **f**), lipid:amide I ratio (**c**, **g**) and carbohydrate:amide I ratio (**d**, **h**) values derived from FT-IR spectra, of wild type *Chlamydomonas reinhardtii* grown under different concentrations of N (**a**–**d**) and P (**e**–**g**) for 7 days. All data are mean ± SE of 3 biological replicates. *Asterisks* denote significant difference compared to control (7 mM P or 1 mM N) treatments

Initial baseline screening was performed on wild type *C. reinhardtii* to examine and compare the metabolic responses to five decreasing concentrations of P (from 1 mM to 1 µM) or N (from 7 mM to 0.07 mM) at late exponential phase (day seven). As seen previously, reduced nutrient availability led to a reduction in biomass production, with a consistent step-by-step decrease in cell biomass as N concentration decreased until there was very little cell growth at 0.07 mM N (a 92 % decrease) (Fig. 1a). The exception was the 3.5 mM N treatment, which stimulated biomass production. A reduction in P concentration to 0.1 mM P did not cause any significant change in growth, while a reduction to 0.05 mM P started to inhibit growth slightly, although not significantly, suggesting mild-to-moderate P limitation, but biomass production was very significantly reduced following 10 µM and 1 µM P treatment (a 56 and 87 % decrease, respectively) (Fig. 1e). Chlorophyll fluorescence (*F*_v_/*F*_m_ ratio) was measured as an indicator of stress and physiological status of the cell. The *F*_v_/*F*_m_ ratio profile for both P and N limitation treatment was equivalent to the biomass production profile, with a significant reduction in *F*_v_/*F*_m_ ratio in response to severe P or N limitation but severe N limitation appeared to be more stressful to the cells (Fig. 1b, f). Following analysis by FT-IR spectroscopy over the wavenumber range of 1780–950 cm^−1^, clear variation was apparent in the baseline-corrected spectra from cells exposed to the different P and N treatments (Supplementary Fig. 1). The strong peak at ~1740 cm^−1^ visible in spectra from both P and N limited cells is indicative of an increase in total lipid whereas overlapped bands between ~1160 and 1036 cm^−1^ are indicative of carbohydrate increases (Dean et al. 2010; Stehfest et al. 2005). These spectral changes indicated that total lipid and carbohydrate responses were the most pronounced following severe N and P limitation. Quantification of total lipid, and carbohydrate band heights, were normalised by expressing these as a ratio to the amide I band (1655 cm^−1^). There was increasing accumulation of lipid and carbohydrate in response to 0.7, 0.3 and 0.07 N (Fig. 1c, d) and in response to 1 and 10 µM P (Fig. 1g–h) but the N-limited cells clearly accumulated more lipid and carbohydrate than any of the P-limited cells. For example, the carbohydrate:amide I ratio value increased from 0.19 in 1 mM P/7 mM N cells to 2.76 in 1 µM P cells (14.5-fold increase) but increased to 9.14 in 0.07 mM N cells (48.1-fold increase), while the lipid:amide I ratio value increased from 0.16 in 1 mM P/7 mM N cells to 0.83 in 1 µM P cells (5.2-fold increase) but increased to 3.87 in 0.07 mM N cells (24.2-fold increase).

**Fig. 2:**
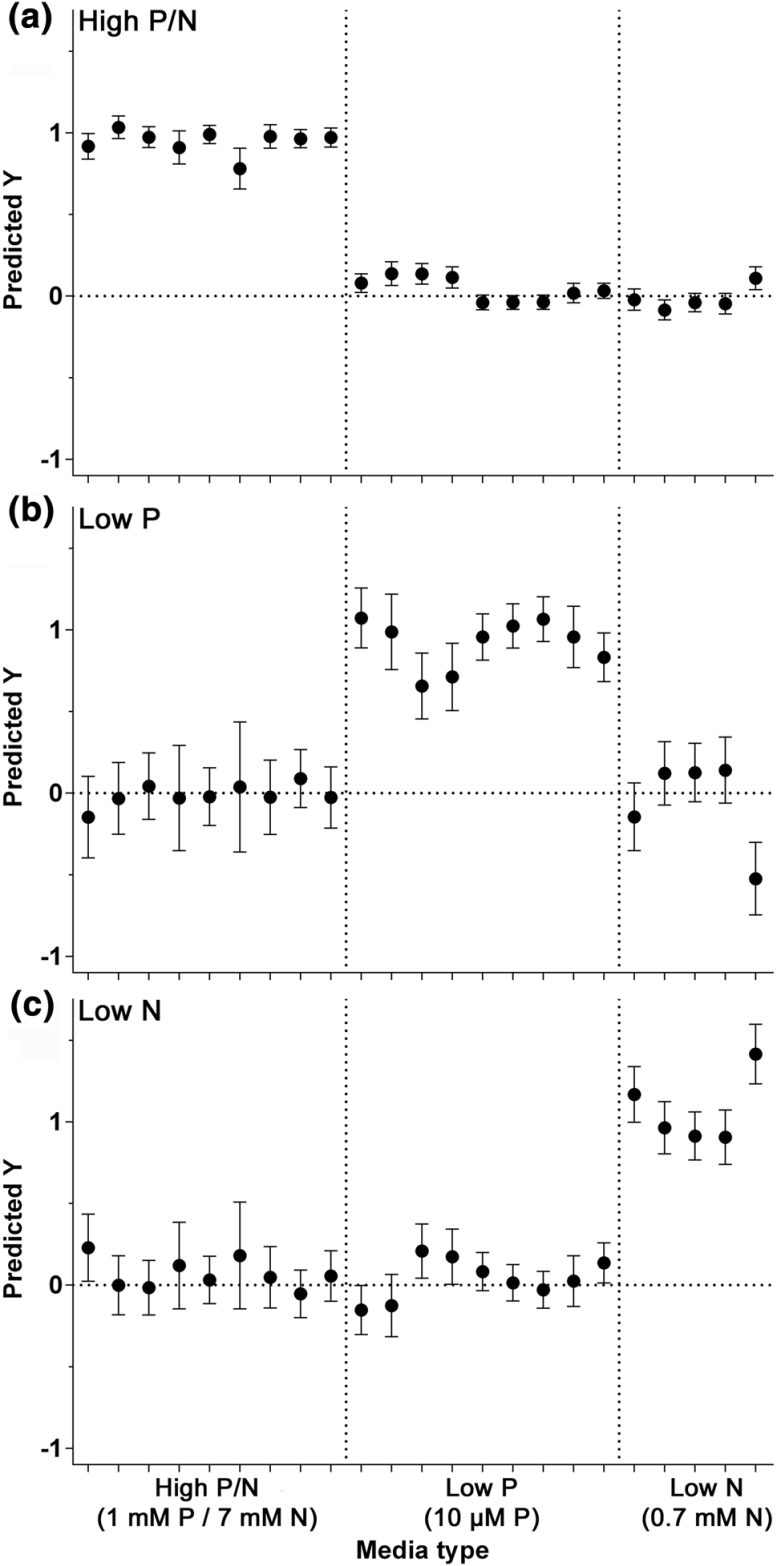
Partial least squares discriminant analysis (PLS-DA) prediction results of non-stressed (high P and high N) grown wild type in comparison to cells cultured in response to P limitation (low P) and N limitation (low N) at day-7 of growth. Ability of PLS-DA linear regression models trained using replicate spectra to predict the identification of high P/N spectra (**a**), low P spectra (**b**) and low N spectra (**c**). The predicted Y values represent a yes (1) or no (0) classification decision for each replicate sample (an average of three technical replicates) in the validation set. *Error bars* indicate 95 % confidence interval around each predicted Y value. Training and validation data consisted of independent biological replicates, and each dataset contained 9 control TAP replicates, 9 low P replicates and 5 low N replicates

While the lowest concentrations of P and N induced the most marked metabolic changes in the cells, with respect to high lipid and carbohydrate accumulation, the fresh weight biomass yields from these treatments were extremely low; 0.91 and 0.54 mg mL^−1^ for the P and N starved cultures, respectively. In contrast, significant increases in lipid and carbohydrate were still observed in the 10 µM P and 0.7 mM N treated cells, but with sufficient biomass. These two nutrient stress conditions (referred to as low P and low N) were then used to examine whether cells exposed to specific stress conditions could be predicted and identified from control (non-stressed) strains using the FT-IR spectral data. To classify stressed and non-stressed strains, a PLS-DA statistical analysis was used to develop predictive models of variation between the strains. The model was generated using a training set of replicate spectra derived from nine control and low P samples and five low N samples (Supplementary Fig. 2). These models used three factors for prediction which accounted for 97 % of the total explained variance. An equal number of test spectra were then evaluated using the PLS-DA model. The Y values of 1 or 0 were set as a yes or no decision as to whether or not a sample belongs to the assigned class with a value of 0.5 as a decision borderline. The model was able to predict accurately strains grown in nutrient replete medium and distinguish them from low P and low N strains (Fig. 2a). Furthermore, despite both nutrient stresses being equivalent in ability to induce lipid and carbohydrate induction, the model was able to accurately distinguish low P strains (Fig. 2b) and low N strains (Fig. 2c) from the rest of the strains. There appears to be differences in the amount of lipid and starch synthesized in response to 0.7 mM N compared to 10 µM P (Fig. 1), which may partly explain the ability of the model to differentiate the N and P stressed cells. However, this might also suggest that other metabolic responses differ between the two nutrient treatments in addition to just lipid and carbohydrate. This has been indicated in previous *C. reinhardtii* metabolomic studies. For example, a GC–MS analysis demonstrated that N limited cells are metabolically distinct from P limited cells and each treatment gives rise to specific changes in distinct amino acids, organic acids and sugars (Bolling and Fiehn 2005). Notably, the lysine biosynthesis metabolite 2-amino-adipic acid increased 9-fold in N limited cells, while tryptophan increased nearly 5-fold, but many metabolites decreased significantly following N limitation including a number of amino acids, fumarate, glucose and malate. In contrast, P limitation induced a 25-fold increase in cysteine concentration, and 4-fold increases in citrate and glycerate, while there were relatively few decreases of amino acids. Likewise, there are clear transcriptional distinctions between P and N limitation, with just a ~5 % similarity in transcriptional response observed between the two stress treatments (Schmollinger et al. 2014). For example, there were no transcript changes related to photosynthetic function that were common between the P and N limitation conditions.

### Screening of *C. reinhardtii* mutants by FT-IR spectroscopy

Wild type *C. reinhardtii* and nine mutant strains were grown until day seven under identical conditions of either sufficient nutrient concentrations (1 mM P, high P or 7 mM N, high N) or limited nutrient concentrations (10 µM P, low P or 0.7 mM N, low N). The mutant lines (Supplementary Table 1) comprised four single gene mutants, including *psr1*, *snrk2.1*, and two alleles of *snrk2.2* (referred to here as *snrk2.2*-*1* and *snrk2.2*-*2*), four double gene mutants (*psr1 snrk2.1*, *psr1 snrk2.2*-*1*, *psr1 snrk2.2*-*2* and *snrk2.1 snrk2.2*-*2*) and one triple mutant (*psr1 snrk2.1 snrk2.2*-*1*). Growth responses were determined by measuring wet biomass, total chlorophyll concentration and *F*_v_/*F*_m_ ratio (Supplementary Fig. 3). All mutant strains responded to N limitation in the same manner as wild type, as seen by significant reduction in biomass, down from ~5–6 to ~2 mg mL^−1^; specifically a 65 % decrease for wild type and ranging from a 59 to 79 % decrease in the mutant strains. In low P conditions, the 68 % biomass reduction in wild type was consistent with each of the *snrk* single and double mutant strains, while all of the strains with a mutation in *PSR1* showed a 77–92 % reduction in biomass, down to 0.45 mg mL^−1^ in the *psr1 snrk2.1 snrk2.2* triple mutant. A significant reduction in total chlorophyll concentration (>90 % decrease) and *F*_v_/*F*_m_ ratio (a 45–91 % decrease) was also consistent in each of the N-limited strains, as expected for a nutrient limitation treatment which is known to cause significant chlorosis. A reduction in total chlorophyll in response to P limitation was consistent in most of the strains (a decrease ranging from 48 to 80 %) apart from *snrk2.1 snrk2.2*-*1* and *psr1 snrk2.1* where there was no significant difference compared to wild type (Supplementary Fig. 3b). However, a change in *F*_v_/*F*_m_ ratio in response to P limitation was inconsistent between the strains and all mutants with a *psr1* genotype showed greatly reduced *F*_v_/*F*_m_ ratio, down to 0.07 from a value of ~0.7 under high P conditions, and thus had reduced photosynthetic efficiency (Supplementary Fig. 3c), suggesting that the cells were experiencing stress in response to P limitation more than the wild type and the *snrk2.1* and *snrk2.2* mutants.

**Fig. 3:**
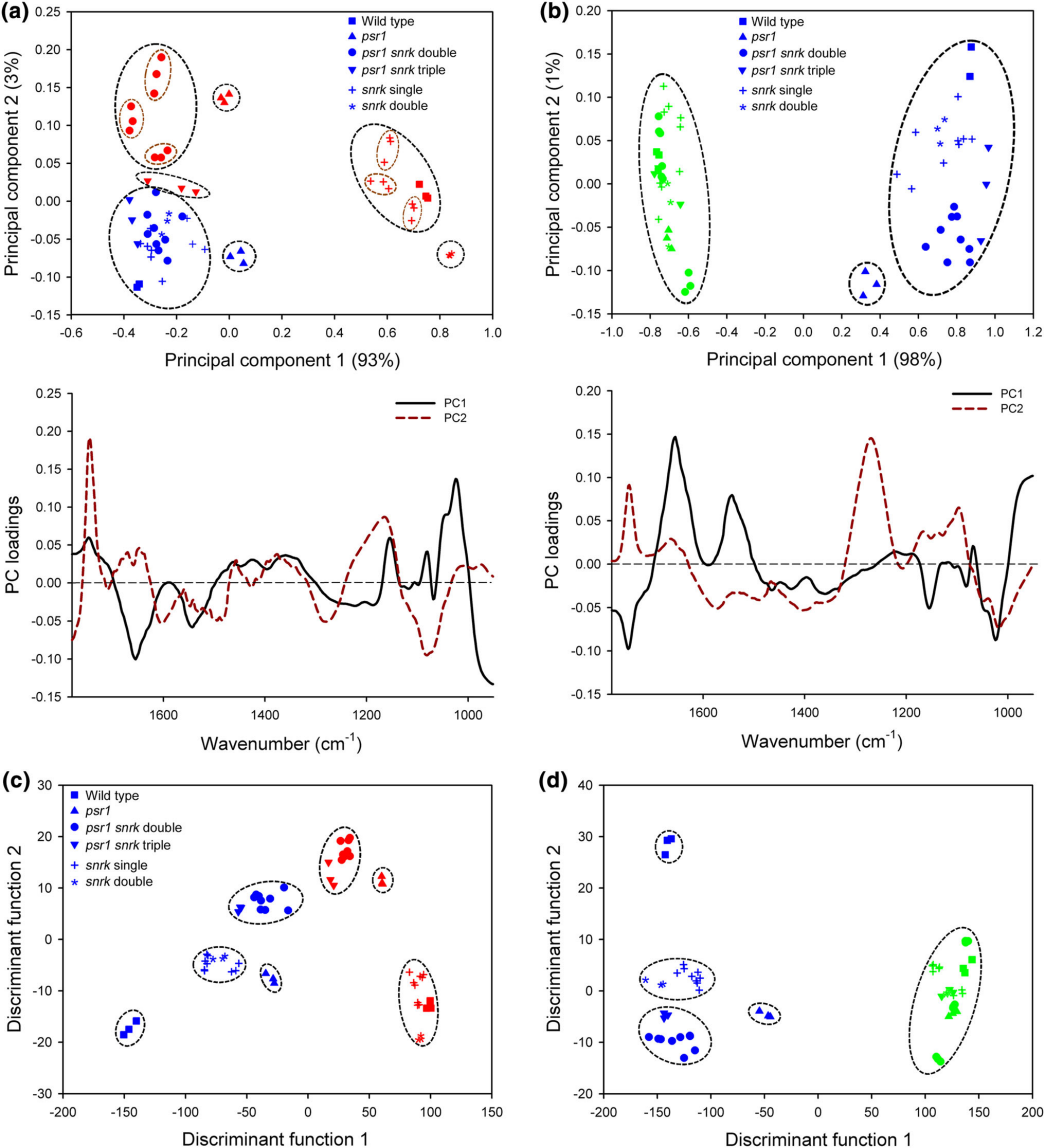
FT-IR spectroscopy screening of wild type and mutant strains in response to P and N limitation. Principal component analysis (PCA) (**a**, **b**) and PC-discriminant function analysis (PC-DFA) (**c**, **d**) of FT-IR spectra (1780–950 cm^−1^) derived from strains cultured in replete or limited concentrations of P (high or low P), indicated by *blue symbols* and *red symbols*, respectively (**a**, **c**), and in replete or limited concentrations of N (high or low N), indicated by *blue symbols* and *green symbols*, respectively (**b**, **d**). Each *symbol* represents the average of 3 technical replicates per biological sample. Different *symbols* each represent 3 biological replicates of each wild type and mutant strain. For this plot *snrk2.1* and *snrk2.2* have been categorized together as ‘*snrk*’. PCA loading plots of PC1 (*bold*) and PC2 (*dashed*) corresponding to high and low P (**a**) and high and low N (**b**) data are shown below the PCA scores plots (Color figure online)

FT-IR spectra were collected from triplicates of all strains under all three growth conditions and analysed by PCA separately for P and N limitation. For clarity in the presented PCA plots, all of the *snrk2.1* and *snrk2.2* mutants are categorised together and indicated by identical symbols (e.g. all nine *snrk2.1*, *snrk2.2*-*1* and *snrk2.2*-*2* single mutant samples are indicated by crosses; *snrk* single mutants, while all nine *psr1 snrk2.1*, *psr1 snrk2.2*-*1* and *psr1 snrk2.2*-*2* double mutant samples are indicated by circles; *psr1 snrk* double mutants). Under high P conditions (blue symbols) the spectra from most of the mutants clustered close with the wild type, although the *psr1* single mutant was slightly separated along PC1 (blue triangles) (Fig. 3a). However, under low P conditions many of the different mutant backgrounds were clearly separated. The spectra from the low P wild type (red squares) were clearly separated from high P wild type (blue squares) along PC1, with this PC determined in part by increased peaks particularly at wavenumbers ~1050 and 1036 cm^−1^, and the *snrk2.1* and *snrk2.2* single mutants showed an equivalent low-P response and were grouped with low P wild type. The *snrk2.1 snrk2.2*-*1* double mutant strains also separated from the high P strains along PC1 but were also clustered away from wild type and *snrk2.1* and *snrk2.2* single mutants. All of the strains with a *psr1* mutant background under low P conditions clustered very differently to low P wild type and were identical to the high P strains on the basis of PC1, but could still be distinguished from high P strains on the basis of PC2, determined partly by an increase in the peak at wavenumber ~1740 cm^−1^ and no increase in peaks at ~1160–1036 cm^−1^ (Fig. 3a). The single *psr1* mutant (red triangles) was again separated from the other strains. However, the ability to discriminate the wild type and mutant strains was markedly reduced under N limitation conditions. All low N strains showed a clear separation with the high N strains on the basis of PC1 (partly determined by increases at wavenumbers ~1740, 1050 and 1036 cm^−1^, and decreases at wavenumbers ~1655 and 1545 cm^−1^) but there was less clear-cut separation amongst the low N strains (green symbols) along PC2 (Fig. 3b). Interestingly, the *psr1 snrk2.2*-*1* double mutant (green circles) could be distinguished from wild type and other mutant strains.

### Supervised statistical methods allow differentiation and prediction of mutants from FT-IR spectra

Further evaluation of the vibrational spectroscopic data was performed using the supervised statistical clustering method, PC-DFA to assess whether additional discrimination of the mutant strains could be achieved. A similar trend seen by PCA was observed by PC-DFA but with better clustering and separation amongst the mutants. PC-DFA is a powerful technique and was able to discriminate the mutants clearly from wild type in non-stressed nutrient replete conditions, but also distinguish the *psr1* single mutant from *psr1 snrk* mutants and from *snrk* single or double mutants (Fig. 3c, d). In the case of the low P treatment (red symbols), the wild type could not be distinguished from the mutants but clustered together with the *snrk* single and double mutant, whereas the *psr1 snrk* double and triple mutants clustered close to the *psr1* single mutant (Fig. 3c). The separation between the mutant strains was reduced in low N treatment and only the *psr1 snrk2.2*-*1* double mutant was clearly separated (Fig. 3d).

**Fig. 4:**
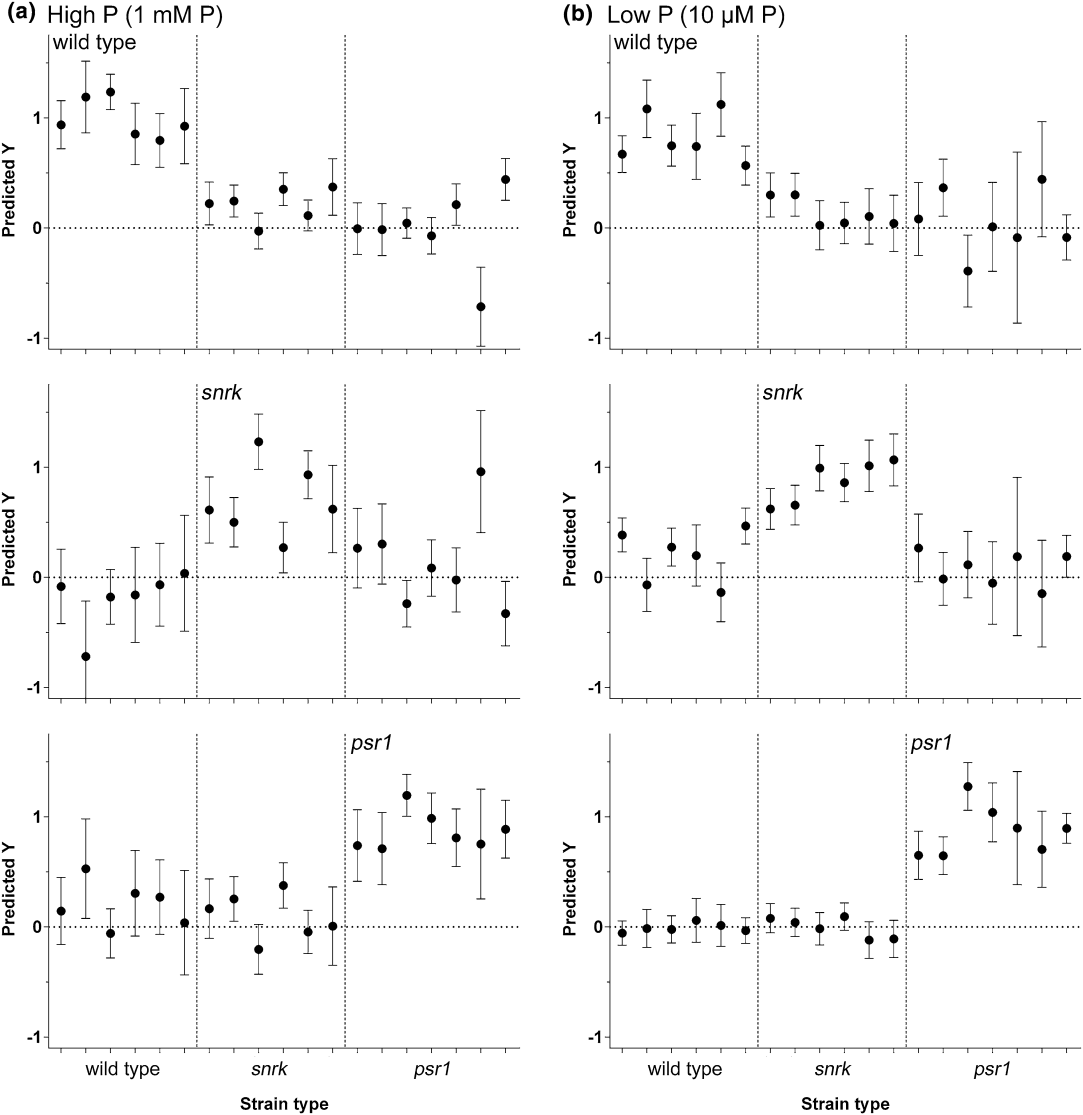
Partial least squares discriminant analysis (PLS-DA) prediction results of wild type (wild type strains) in comparison to mutant strains containing the *snrk2.1* and/or *snrk2.2* mutation but not the *psr1* mutation (*snrk* type strains), or the *psr1* mutation either alone or in combination with *snrk2.1*/*snrk2.2* mutation (*psr1* type strains). Ability of PLS-DA linear regression models trained using replicate spectra to predict the identification of wild type, *snrk* and *psr1* strains either under high P (**a**) or low P (**b**) conditions. The predicted Y values represent a yes (1) or no (0) classification decision for each replicate sample (an average of three technical replicates) in the validation set. *Error bars* indicate the 95 % confidence interval around each predicted Y value. Training and validation data consisted of independent biological replicates, and each dataset contained 6 wild type replicates, 6 *snrk* strain replicates and 7 *psr1* strain replicates

PLS-DA modeling was successful at discriminating between wild type grown under different nutrient conditions (Fig. 2) therefore the same approach was examined to attempt to classify the mutant strains and to develop predictive models of variation between the mutants. This was to examine whether genotypic groupings such as the presence or absence of the *psr1* mutation or a *snrk* mutation (either *snrk2.1* or *snrk2.2*) could be predicted from the FT-IR spectra. Strains were divided into three types; wild type, those containing the *psr1* mutation (*psr1* type) and those containing the *snrk2.1* and/or the *snrk2.2* mutation but not the *psr1* mutation (*snrk* type). The model was generated using a training set of replicate spectra derived from six wild type, six *snrk* and eight *psr1* samples grown under either P replete or P limited conditions (Supplementary Fig. 4). The quality of these models is shown in Fig. 4, showing predicted versus measured plots of the calibration and cross-validation based on FT-IR. These models used three factors for prediction which accounted for 99 % of the total explained variance. Initially the FT-IR data were split into two sets of replicate spectra, a calibration set and an external validation set both containing all strains (wild type and all mutants). The high P model was able to clearly predict and differentiate wild type from the mutant strains, and *psr1* from wild type and *snrk* strains, although less robustly compared to the wild type classification. In contrast, the *snrk* prediction was weak under this condition. The low P prediction models found that all *psr1* mutation-containing strains could be clearly differentiated from wild type and *snrk* mutants, and there was also reasonable classification of wild type strains and *snrk* strains (Fig. 4).

The use of FT-IR spectroscopy for discriminating microorganisms has been previously demonstrated, particularly for identification and discrimination between species of bacteria, cyanobacteria or fungal species (Bounphanmy et al. 2010; Timmins et al. 1998; Winder et al. 2004). However, discrimination between eukaryotic microalgae species has been more challenging compared to non-photosynthetic microorganisms like yeast (Domenighini and Giordano 2009; Driver et al. 2015). Possibly this is because microalgae are metabolically complex organisms that can utilize both photo-autotrophic and photo-heterotrophic lifestyles, can synthesise a diverse array of metabolites but can also show considerable phenotypic plasticity in their metabolic responses to environmental changes. As a result the same microalgal species can produce very different FT-IR spectra under different cultivation or stress conditions (e.g. Supplementary Fig. 1). It is probably this subtle metabolic variation between single mutations that allows the ability to discriminate, as long as the strains are grown under equivalent, controlled conditions. We have shown here that single mutants can be clearly differentiated and classified using FT-IR spectral information and multivariate statistical methods. However, mutants of some genes that appear to play a significant role in metabolic regulation, such as *psr1*, clearly are more distinct and show a more pronounced FT-IR phenotype than other mutants, like a single *snrk2.1* or *snrk2.2* mutant. These have a much more subtle phenotype and are less easily distinguished, even when using supervised chemometric analysis methods.

### Lipid profiling of mutants by UHPLC–MS

**Fig. 5:**
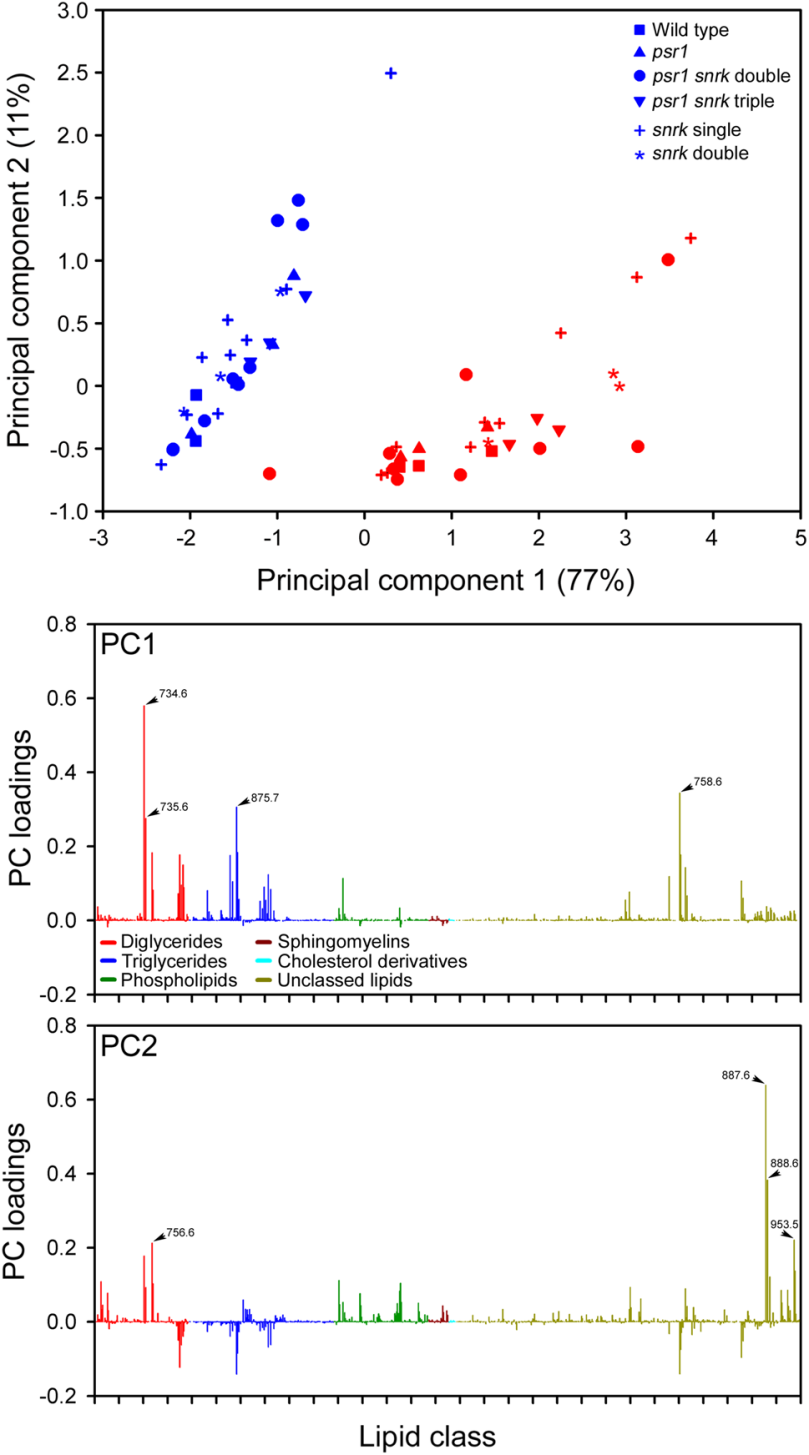
LC–MS analysis of wild type and mutant strains in response to P limitation. Principal component analysis (PCA) of UHPLC–MS spectra derived from wild type and mutant strains cultured in replete concentrations of P (1 mM), indicated by *blue symbols*, and in limited concentrations of P (10 µM), indicated by *red symbols*. Different *symbols* represent the different wild type and mutant strains. For this plot all *snrk2.1* and *snrk2.2* have been categorized together as ‘*snrk*’. PCA loading plots of PC1 and PC2 are shown below the scores plot. Peaks have been categorized into one of five lipid classes as indicated by *peak colour*, and within each class are arranged in ascending *m*/*z* value. Multiple phospholipid types (described in Supplementary Fig. 7) are grouped together as phospholipids. Peaks with a PC loading value greater than 0.2 are highlighted and *m*/*z* value indicated. Lipid peak definitions are shown in Supplementary Table 2 (Color figure online)

**Fig. 6:**
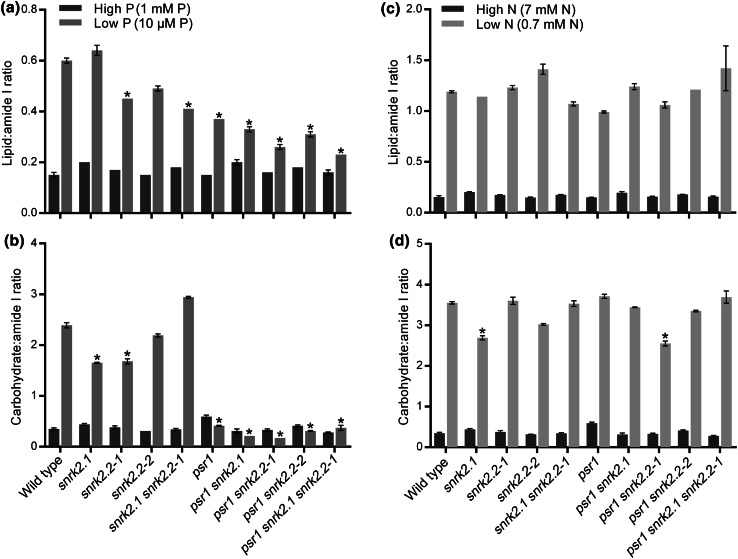
Lipid and carbohydrate quantification from FT-IR spectroscopy screening of wild type and mutant strains in response to P and N limitation. Mean lipid:amide I ratio (**a**, **c**) and carbohydrate:amide I ratio (**b**, **d**) values (±SE) derived from 3 FT-IR spectra (1780–950 cm^−1^) from each wild type and mutant strain under high and low P (**a**, **b**) and high and low N (**c**, **d**) conditions. Each spectrum is an average of three technical replicates. *Asterisks* denote significant difference compared to the control (high P or N) treatment (Color figure online)

To compare the high-throughput FT-IR spectroscopy profiling of the strains with an alternative analytical method, UHPLC–MS was performed on a non-polar lipophilic fraction isolated from wild type and mutant strains grown under high P and low P conditions until day seven. PCA of the UHPLC–MS data showed some similarity with the profile seen by PCA of the FT-IR spectra. There was clear separation of the high P and low P treated strains on the basis of PC1 (determined largely by increases in peaks classified as diglycerides and triglycerides), but for either treatment group, further clear separation on the basis of genetic background was lacking (Fig. 5). Even treatment of the data by PC-DFA was unable to distinguish many of the mutant lines apart from discrimination of *snrk2.1* and *psr1 snrk2.2*-*2* from the other strains on the basis of DF2, determined in part by changes in peaks classified as triglycerides (Supplementary Fig. 5). In particular, it was not possible to distinguish *psr1* mutants from wild type under P limitation conditions, which was very different from the outcome using FT-IR data. The explanation for this is likely to be due to the more limited metabolic information gained from the UHPLC–MS analysis of the non-polar fractions in contrast to the macromolecular quantification of whole cells by FT-IR spectroscopy, which identified significant changes in carbohydrates (Fig. 6b), particularly starch (Supplementary Fig. 6). This difference in the information content between different metabolomics methods has been recently illustrated for a set of bacteria that displayed different metabolic diversities and virulence factors (AlRabiah et al. 2014). Furthermore, the majority of the metabolites determined by UHPLC–MS are lipids (Supplementary Fig. 7; Supplementary Table 2), and while there were some fairly subtle differences in total lipid accumulation between wild type and *psr1* in response to P limitation, the most marked change was for the accumulation of starch (see below).

A number of methods have been developed for screening and examination of microalgae strains for metabolite phenotypes, particularly for high lipid-yielding strains. These have often included the use of chromatography or lipid stains such as Nile red combined with fluorescent sorting. Whilst these methods have been shown to be successful for fairly rapid screening of novel lipid metabolism mutants (Cagnon et al. 2013; Terashima et al. 2015), a limitation is that only one metabolic parameter at a time is being determined. These results demonstrate one of the clear benefits of the FT-IR spectroscopy-based screening method over other approaches in that it provides the ability to screen multiple metabolic characteristics simultaneously. Furthermore, the more complex metabolic fingerprint generated by the FT-IR spectra allows better discrimination of mutations (e.g. Figs. 3 vs 5). However, for a subsequent detailed characterization of a particular metabolite class, such as TAG fatty acid composition in a lipid fraction, a more focused method such as LC–MS is then necessary to obtain a level of information that FT-IR or fluorescent lipid stain analysis would not alone provide. Nevertheless, we show that the FT-IR methodology described provides a suitable first-pass analysis and pre-screening of strains before more detailed and time-consuming characterization is performed.

### Metabolic changes underlying the mutant discrimination

Metabolic differences from the FT-IR spectra were examined further in order to attempt to explain some of the distinctions observed between the mutants. As seen in the initial experiments, the loading plots of the low P and low N treatments indicated that the differences between nutrient sufficiency and limitation for the wild type and wild type-clustered strains was due largely to increased accumulation of lipids and carbohydrates (Fig. 3). This was particularly clear in all of the low N treated strains where a consistent increase in lipid:amide I and carbohydrate:amide I ratio values compared to the high-N treated strains were seen, ranging from 6.1- to 9.5-fold increases in lipid:amide I ratio and 6.2- to 13.2-fold increases in carbohydrate:amide I ratio (Fig. 6c, d). Likewise, in the low P experiments a significant increase in lipid and carbohydrate was indicated in the wild type and *snrk2.1* and *snrk2.2* single and double mutants in response to P limitation, ranging from 2.3- to 4.0-fold increases in lipid:amide I ratio and 3.8- to 8.6-fold increases in carbohydrate:amide I ratio (Fig. 6a, b). However, for the mutants possessing a *psr1* genotype (*psr1*, *psr1 snrk2.1*, *psr1 snrk2.2*, and *psr1 snrk2.1 snrk2.2*), the distinction from the wild type and *snrk2.1*/*snrk*2.2 strains appeared to be due to a lack of carbohydrate accumulation in response to P limitation (no significant increase in carbohydrate:amide I ratio in any low P grown *psr1* genotype cells), and thus having a carbohydrate phenotype that was identical to the high P treated cells (Fig. 6b). The lipid:amide I ratio values in these *psr1*-containing mutants in low P was inhibited relative to wild type (ranging from 1.4- to 2.5-fold increases in lipid:amide I ratio), but the increase relative to high P cells was not completely abolished (Fig. 6a), thus the low P *psr1* mutants were still distinct from the high P cells in the PCA scores plot (Fig. 3a).

As well as providing a demonstration of the ability to screen and profile mutants by FT-IR spectroscopy efficiently, this analysis has provided some more insight into the functions of PSR1, SNRK2.1 and SNRK2.2. Mutation of *PSR1* clearly impacts on starch metabolism and to a lesser extent on lipid metabolism, as we have observed previously (Bajhaiya et al. unpublished), but specifically under P limitation, with no significant metabolic change under N limitation conditions. In contrast, there was no evidence of major metabolic regulation by SNRK2.1 or SNRK2.2 under P or N limitation, or under nutrient replete conditions. SNRK2.1 and SNRK2.2 both play important roles to control the cell’s responses to S limitation (Davies et al. 1999; Gonzalez-Ballester et al. 2008). Although there is no evidence of a direct role for these proteins in P limitation response, there is evidence that S metabolism is altered by P limitation, and in a *psr1* mutant background, more extreme internal P limitation induces greater S limitation, which in turn induces expression of the positive regulator *SNRK2.1* (Moseley et al. 2009). Furthermore, an epistatic relationship between *PSR1* and *SNRK2.2* has been observed such that a *psr1 snrk2.2* mutant is more sensitive to P limitation compared to *psr1* (Moseley et al. 2009). However, we did not observe significant metabolic differences between *psr1* single mutants and *psr1 snrk2.1* or *psr1 snrk2.2* double mutants during P limitation in our analysis. If anything, there were more obvious metabolic differences between these single and double mutants during nutrient replete and N limitation treatments (Fig. 3d). There is some evidence of the *snrk2.1* mutant exhibiting specific transcriptional and metabolic phenotypes during nutrient replete conditions, including alteration in H_2_ biosynthesis and anoxia (Gonzalez-Ballester et al. 2010). However, we suggest that not just *snrk2.1* but all of the mutants studied here cause some metabolic alteration under nutrient replete conditions. Indeed one of the interesting outcomes from this study was the clear distinction observed between wild type strains and all of the mutant strains under nutrient replete conditions, as determined by the specific metabolic profiles discerned by PC-DFA (Fig. 3b, c). A detailed examination of these data sets and further metabolite quantification will be able to explain these phenotypes more clearly in the future.

The changes in total lipid and carbohydrate in the nutrient-limited cells measured by the FT-IR spectra were validated by biochemical methods and confirmed to be due to increased biosynthesis of neutral lipids and starch (Supplementary Figs. 6, 7). For example, in low N conditions, starch accumulated to concentrations between ~40 and 70 µg mg^−1^ fresh weight biomass in all strains compared to a concentration of ~2–4 µg mg^−1^ in nutrient replete conditions. There was a similar increase in starch concentration in low P media for the wild type and *snrk* genotype strains, but starch concentration was very low for all of the strain possessing the *psr1* mutation (15.4 µg mg^−1^ for *psr1*; 6.3 µg mg^−1^ for *psr1 snrk2.1*; 2.6 µg mg^−1^ for *psr1 snrk2.2*-*1*; 27.4 µg mg^−1^ for *psr1 snrk2.2*-*2*; 2.9 µg mg^−1^ for *psr1 snrk2.1 snrk2.2*-*1*). Total protein was also quantified and its concentration was relatively constant under all treatments across all of the lines, ranging from 1.3 to 2.3 µg mg^−1^ fresh weight biomass (mean of 1.9 ± 0.1 µg mg^−1^) for high P/N, from 0.9 to 2.6 µg mg^−1^ (mean of 1.7 ± 0.2 µg mg^−1^) for low P, and from 1.3 to 2.4 µg mg^−1^ (mean of 1.7 ± 0.1 µg mg^−1^) for low N (Supplementary Fig. 6c, f), indicating the suitability of using amide I for normalisation of the FT-IR spectra bands.

Accumulation of neutral lipid was indicated by Nile red staining and gave a profile that was equivalent to that determined by FT-IR spectroscopy. In low N treatments, all strains displayed concentrations of neutral lipid ranging from ~35 to 80 µg mg^−1^ fresh weight biomass compared to a concentration of ~1.5–2.5 µg mg^−1^ in high nutrient medium. In low P treatments neutral lipid accumulated to ~18–30 µg mg^−1^ in wild type and most mutant strains, but this was inhibited slightly, up to 5–13 µg mg^−1^ in *psr1 snrk2.2*-*1*, *psr1 snrk 2.2*-*2* and *psr1 snrk2.1 snrk2.2*-*1*. UHPLC–MS analysis confirmed that primarily, putatively identified triglycerides increased on average by 2.2-fold in low P treatment, while most classes of phospholipids decreased in low P, notably phosphatidylcholines (an average 1.8-fold decrease), phosphatidylethanolamines (an average 1.6-fold decrease) and phosphatidylserines (an average 1.7-fold decrease), as well as sphingomyelins (an average 1.8-fold decrease) (Supplementary Fig. 7). Although P limitation induced total lipid accumulation in all strains with significant reduction relative to wild type in some of the mutants, at the level of individual lipid classes, most of the mutants showed only subtle differences compared to wild type (Supplementary Fig. 7). Finally, the reliability of the FT-IR spectroscopy-based prediction of lipid and carbohydrate content was indicated by strong linear correlation between lipid:amide I ratio and total lipid, and between carbohydrate:amide I ratio and starch content, with *R*^2^ values >0.8 in both cases (Supplementary Fig. 8).

## Concluding remarks

In conclusion, this study has demonstrated the use of FT-IR spectroscopy for robust but simple and fast metabolite screening of mutants, which could easily be expanded to allow rapid screening of genome-scale mutant collections. Although validated here with *Chlamydomonas*, it will be applicable for any algae species, but also any plant species. It would be appropriate for bioprospecting for novel strains or the identification of novel mutants with metabolic characteristics that may be suitable for a variety of biotechnological applications, both related to biofuel use and other high-value metabolites. It will also provide a suitable first-pass analysis and pre-screen of microalgae mutants before more detailed ‘omics characterisation is performed.

## Electronic supplementary material

Supplementary material 1 (PDF 2022 kb)
