# Supplementary material for: High-throughput metabolic screening of microalgae genetic variation in response to nutrient limitation
Source: Metabolomics. 2015 Nov 13;12:9. doi: 10.1007/s11306-015-0878-4 (PMC4644200; doi:10.1007/s11306-015-0878-4)
Supplement: Supplementary file 1 — Supplementary material 1 (PDF 2022 kb) [file 11306_2015_878_MOESM1_ESM.pdf]

Supplementary information

High-throughput metabolic screening of microalgae genetic variation in response to nutrient limitation

Amit K. Bajhaiya[1,a], Andrew P. Dean[1,2,a], Thomas Driver[1], Drupad K. Trivedi[3], Nicholas J. W. Rattray[3], J. William Allwood[3,4] Royston Goodacre[3], Jon K. Pittman[1]*

[1]Faculty of Life Sciences, The University of Manchester, Michael Smith Building, Oxford Road, Manchester M13 9PT, UK
[2]Department of Geography, University of Sheffield, Sheffield S10 2TN, UK
[3]School of Chemistry, Manchester Institute of Biotechnology, The University of Manchester, 131 Princess Street, Manchester M1 7DN, UK
[4]Environmental & Biochemical Sciences Group, The James Hutton Institute, Invergowrie, Dundee DD2 5DA, Scotland UK

[a]These authors contributed equally to the paper

*Corresponding author: Dr Jon Pittman, Faculty of Life Sciences, University of Manchester, Michael Smith Building, Oxford Road, Manchester M13 9PT, UK; Tel: +44 (0)161 275 5235; Fax: +44 (0)161 275 5082; Email: jon.pittman@manchester.ac.uk

This file includes eight supplementary figures and two supplementary tables:

Supplementary Figure 1. Representative EMSC-corrected FT-IR spectra from wild type cultured in replete and limiting P and N conditions after seven days.

Supplementary Figure 2. PLS scores plot of FT-IR spectra derived from wild type cells cultured in nutrient replete conditions in comparison to cells cultured in response to P limitation and N limitation after seven days of growth.

Supplementary Figure 3. Physiological responses of wild type and mutant strains in response to P and N limitation.

Supplementary Figure 4. PLS scores plot of FT-IR spectra derived from wild type, *snrk2.1* and/or *snrk2.2* mutants and *psr1*-containing mutants (*psr1* type strains).

Supplementary Figure 5. PC-DFA of LC-MS data from wild type and mutant strains in response to P limitation.

Supplementary Figure 6. Biochemical validation of metabolite concentrations in wild type and mutant in response to nutrient replete and limited conditions.

Supplementary Figure 7. Relative changes in lipid composition of wild type and mutant strains determined from UHPLC-MS analysis in positive electrospray ionization mode.

Supplementary Figure 8. Correlation plots of the lipid:amide I ratio or carbohydrate:amide I ratio values determined from FT-IR spectra and the corresponding neutral lipid or starch concentration values determined by biochemical quantification.

Supplementary Table 1. *Chlamydomonas reinhardtii* strains used in this study.

Supplementary Table 2. Matched identifications for each significant lipid peak based on PC and DF loading values derived from PCA and PC-DFA of UHPLC-MS analysis of wild type and mutant strains in response to P limitation.

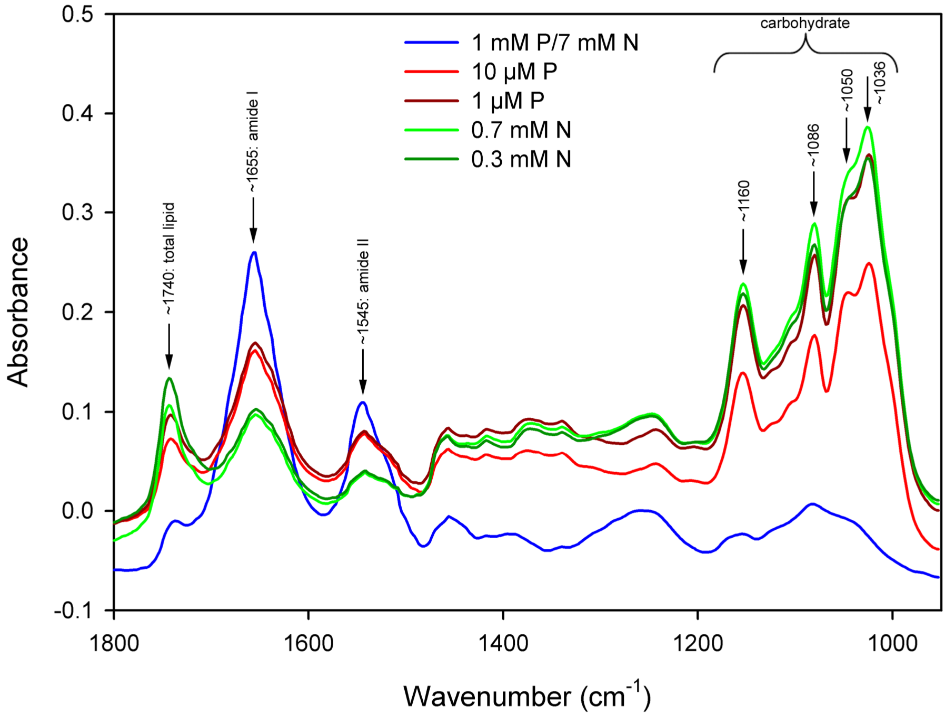

Supplementary Fig. 1 Representative FT-IR spectra in the range of 1780–950 cm^{-1} (EMSC-corrected) from wild type *Chlamydomonas reinhardtii* cultured in high P and N (1 mM P, 7 mM N), and limiting concentrations of P (10 and 1 µM P) and N (0.7 and 0.3 mM N) at day-7. Major lipid, carbohydrate and protein bands are indicated

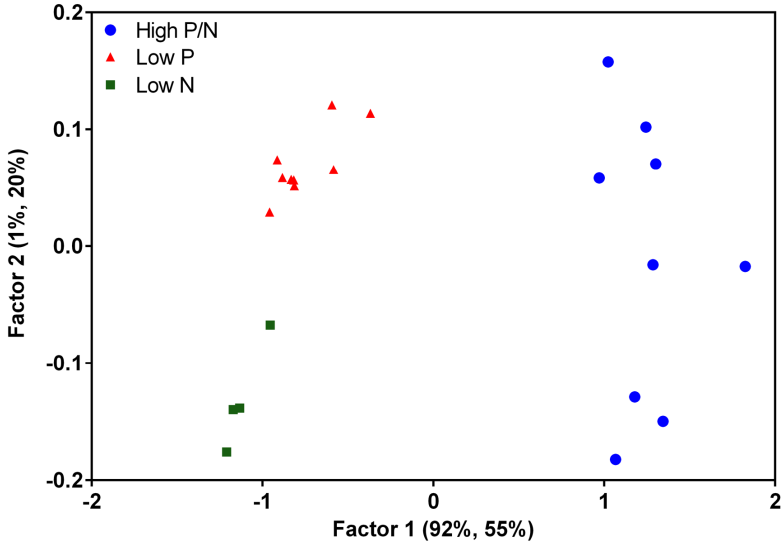

Supplementary Fig. 2 PLS scores plot of FT-IR spectra (1780–950 cm^{-1}) derived from wild type cells cultured in nutrient replete (high P/N, 1 mM P, 7 mM N) conditions in comparison to cells cultured in response to P limitation (low P, 10 µM P) and N limitation (low N, 0.7 mM N) at day-7 of growth. Each symbol represents the average of 3 technical replicates per biological sample. Different colours/symbols represent different nutrient conditions. Plot based on 9 biological replicates for high P/N and low P, and 5 biological replicates for low N

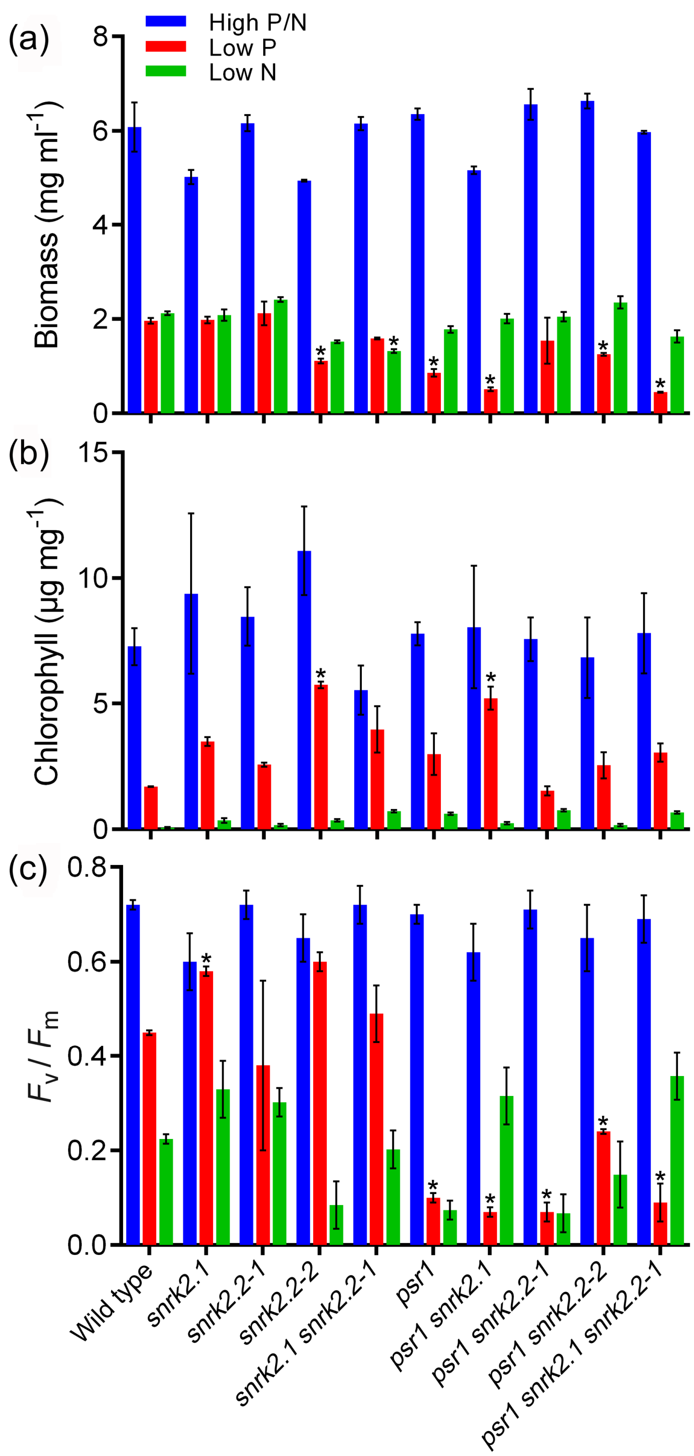

Supplementary Fig. 3 Physiological responses of wild type and mutant strains in response to P and N limitation. **a** – **c** Fresh weight biomass (**a**), total chlorophyll concentration (**b**) and chlorophyll fluorescence (F_v/F_m ratio) (**c**) of wild type and mutant strains under P and N limitation (low P, 10 µM P and low N, 0.7 mM N) in comparison to nutrient replete (high P/N, 1 mM P and 7 mM N) conditions at day 7 of growth. All data points are means ±SE of 3 biological replicates. Asterisks denote significant difference compared to wild type strains

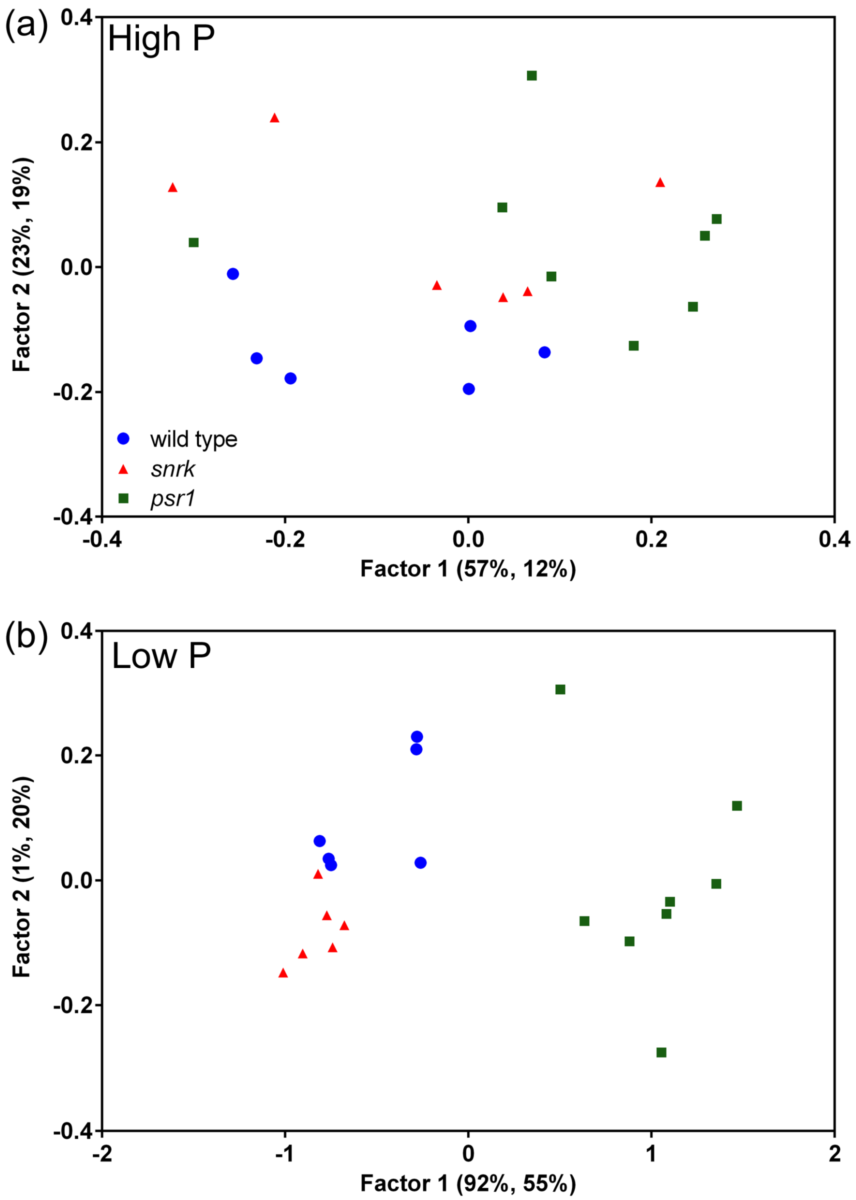

Supplementary Fig. 4 PLS scores plot of FT-IR spectra (1780–950 cm^{-1}) derived from wild type, *snrk2.1* and/or *snrk2.2* mutants (*snrk* type strains) and *psr1*-containing mutants (*psr1* type strains). **a** and **b** Strains cultured in P replete (high P, 1 mM P) conditions (**a**) or P limitation (low P, 10 µM P) conditions (**b**) at day 7 of growth. The spectra deriving from 6 wild type replicates, 6 *snrk* replicates and 8 *psr1* replicates were used as training data to generate PLS-DA linear regression models. Each symbol represents the average of 3 technical replicates per biological sample

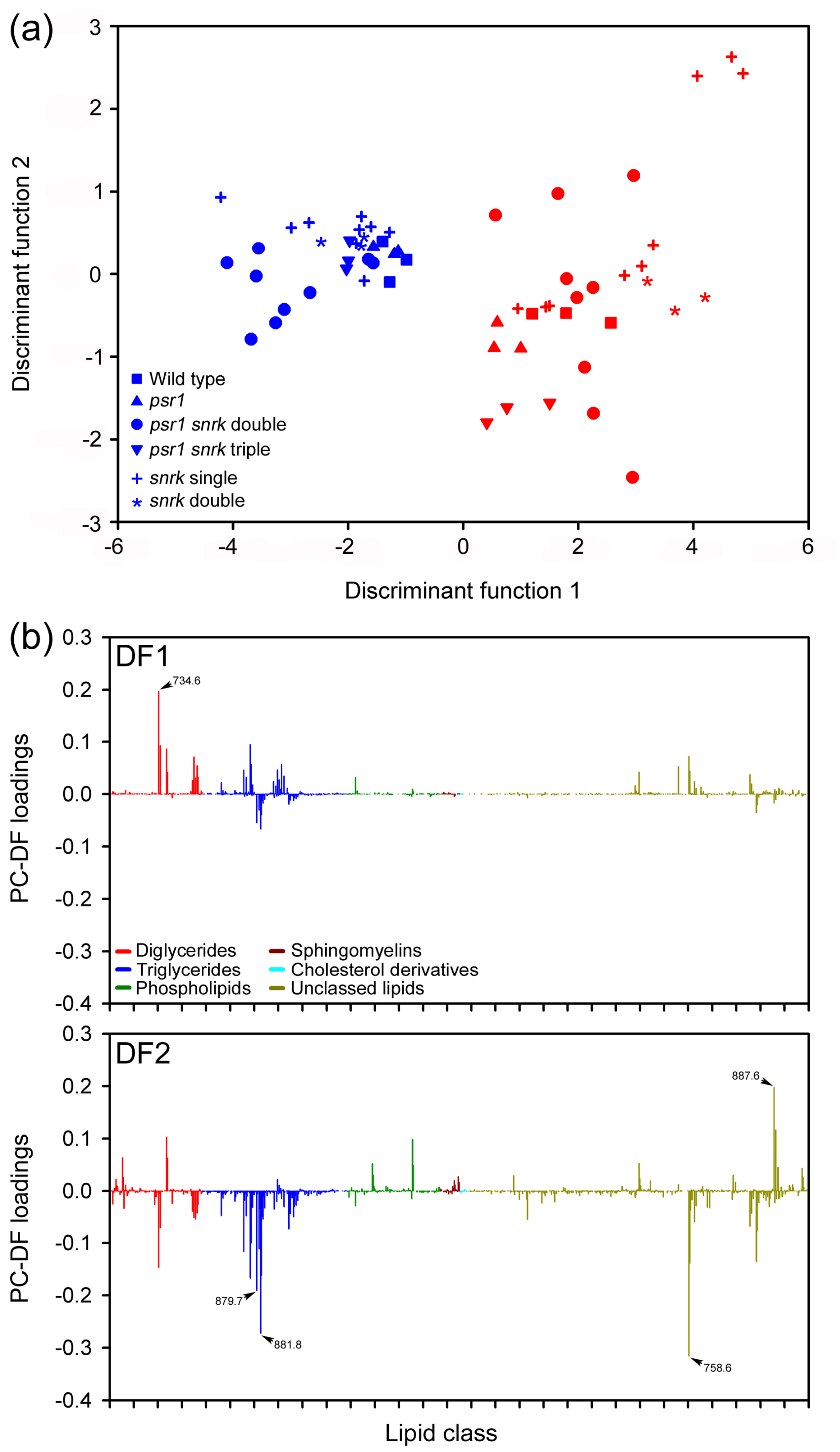

Supplementary Fig. 5 LC-MS analysis of wild type and mutant strains in response to P limitation. **a** PC-discriminant function analysis (PC-DFA) of UHPLC-MS spectra (positive electrospray ion mode) derived from wild type and mutant strains cultured in replete concentrations of P (1 mM), indicated by blue symbols, and in limited concentrations of P (10 µM), indicated by red symbols. Different symbols represent the different wild type and mutant strains. For this plot all *snrk2.1* and *snrk2.2* mutants have been categorized as '*snrk*'. **b** PC-DFA loading plots of DF1 and DF2. Peaks have been categorized into one of five lipid classes as indicated by peak colour, and within each class are arranged in ascending *m/z* value. Multiple phospholipid types (described in Supplemental Fig. 7) are grouped together as phospholipids. Peaks with a loading value greater than 0.2 are highlighted and *m/z* value indicated. Lipid peak definitions are shown in Supplemental Table 2

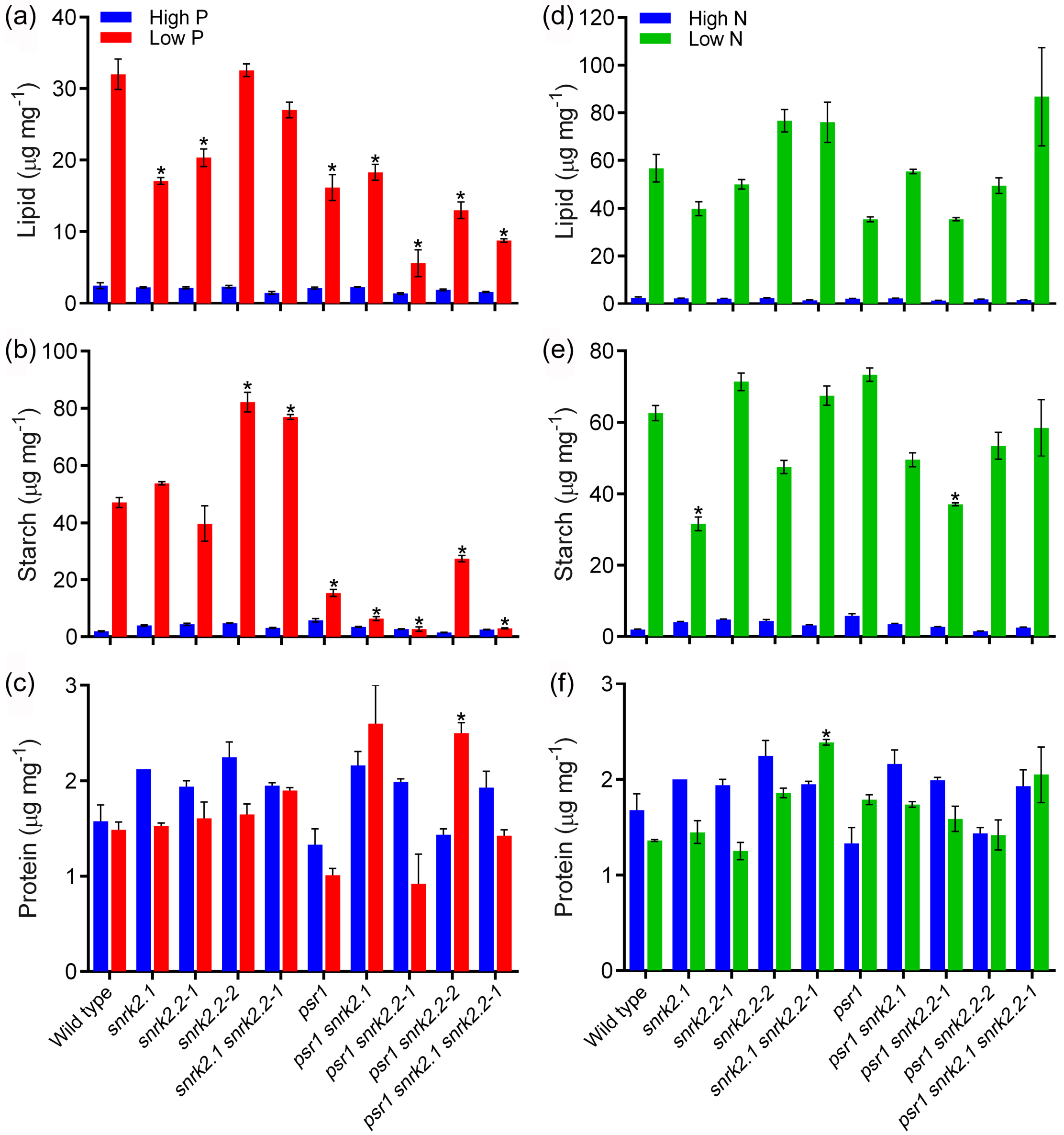

Supplementary Fig. 6 Biochemical validation of metabolite concentrations in wild type and mutants in response to nutrient replete and limited conditions. **a** – **f** Neutral lipid (**a**, **d**), starch (**b**, **e**) and protein (**c**, **f**) concentration of wild type and mutant lines, determined at day 7 in response to nutrient replete treatments (1 mM P, high P and 7 mM N, high N) and nutrient limitation treatment, either low P (10 µM P) (**a** – **c**) or low N (0.7 mM N) (**d** – **f**). All data points are means ±SE of at least 3 biological and 3 technical replicates. Lipids were determined using Nile Red quantification, starch was determined using a Total Starch Assay kit (Megazyme) using the manufacturer's specifications and protein using the Bradford assay

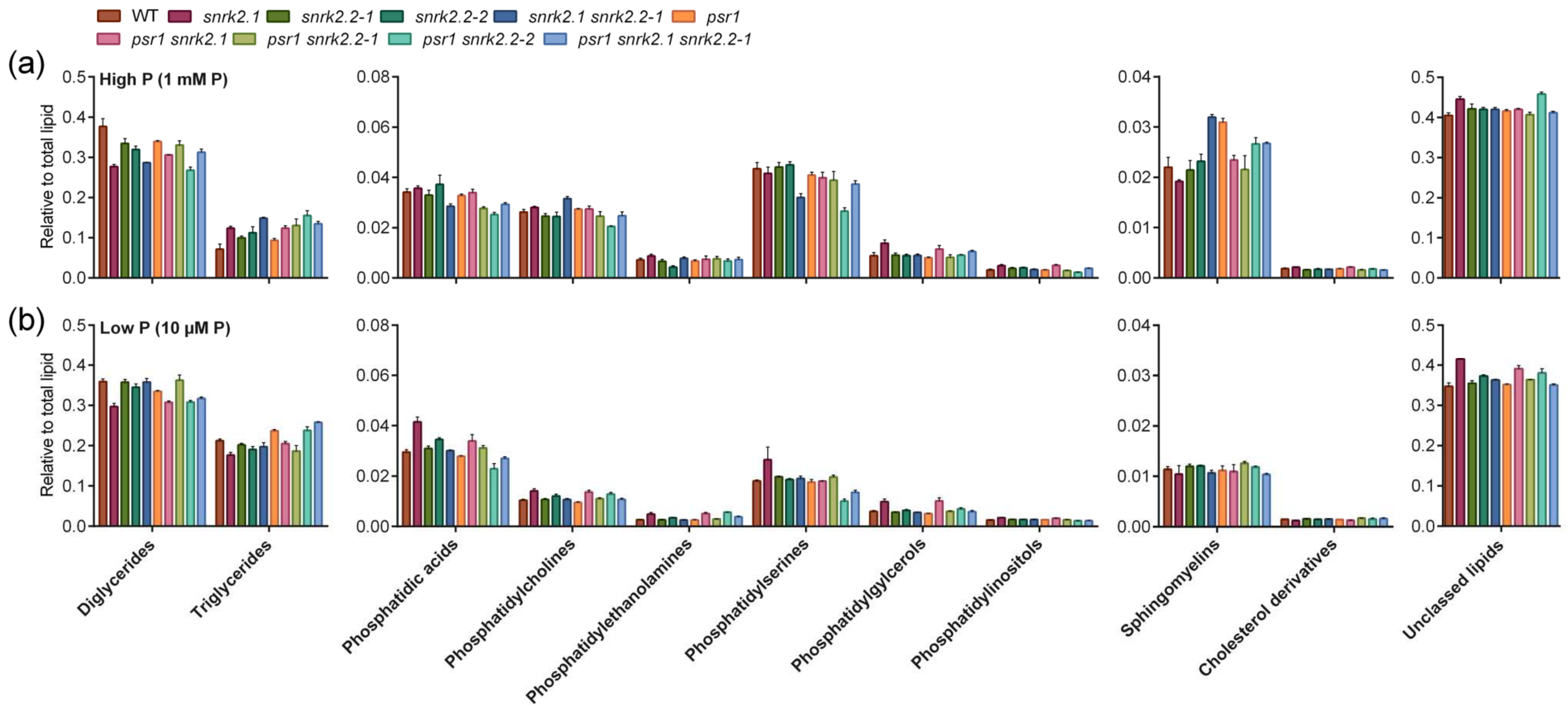

Supplementary Fig. 7 Relative changes in lipid composition of wild type and mutant strains determined from positive ionization mode UHPLC-MS. **a** and **b** Lipid composition in strains grown under P replete (1 mM P) (**a**) and P limitation (10 µM P) (**b**) conditions. Data are means ±SE derived from at least 3 replicate spectra which have been normalized on the basis of fresh weight biomass

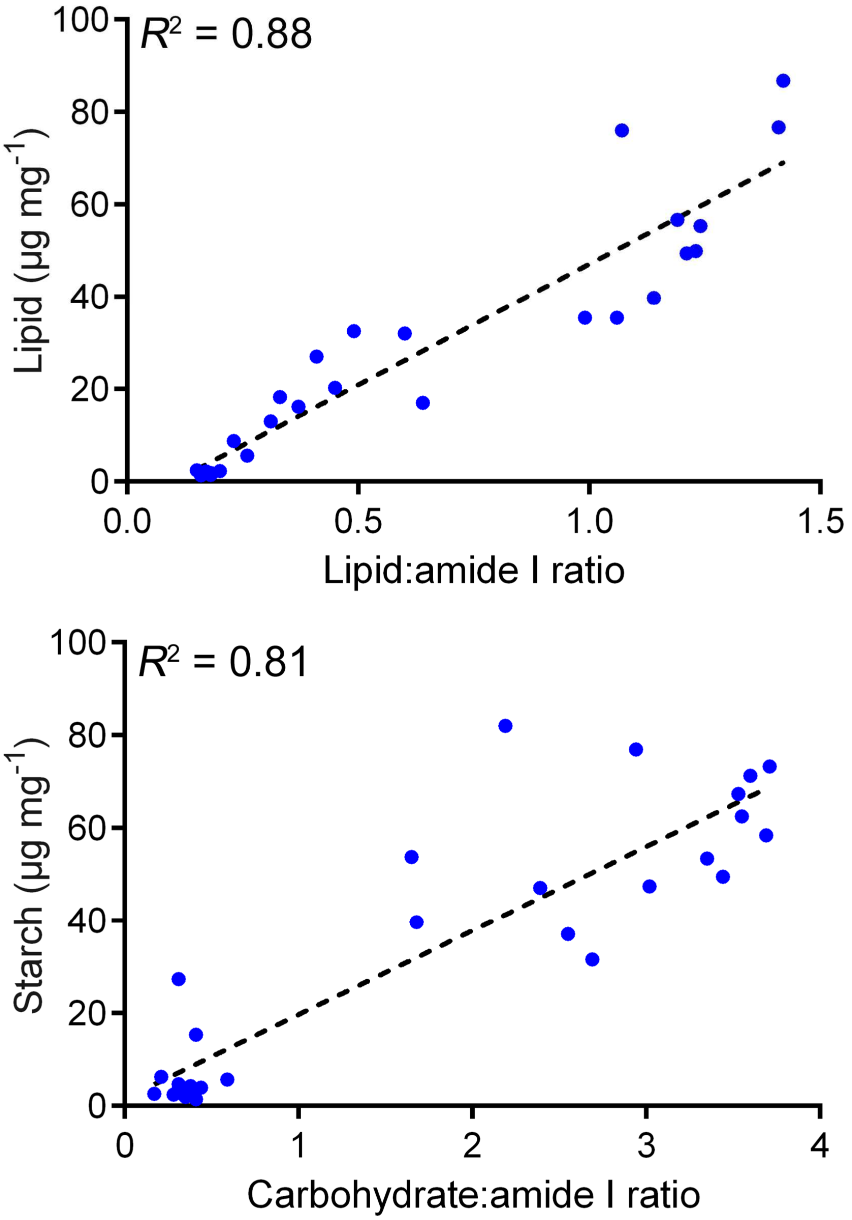

Supplementary Fig. 8 Correlation plots of the lipid/amide I ratio or carbohydrate/amide I ratio values determined from FT-IR spectra and the corresponding neutral lipid or starch concentration values determined by biochemical quantification. Lipids were determined using and Nile Red quantification. Starch was determined using a Total Starch Assay kit (Megazyme) using the manufacturer's specifications. Data are from wild type and mutant strains and from all nutrient treatments. Each data point is a mean value (±SE) of at least three biological and three technical replicates

Supplementary Table 1 *Chlamydomonas reinhardtii* strains used in this study

**Strain ID**[a]	**Name**	**Genotype**	**Source**	**Reference**[b]
CC125 (CCAP11/32C)	Wild type	*nit1nit2cw+mt+*	Culture Collection of Algae and Protozoa	(Pröschold et al. 2005)
CC4267	*psr1*	*psr1-1cw+nit-mt-*	Arthur Grossman (Carnegie Institution)	(Shimogawara et al. 1999)
CC4275	*snrk2.1*	*snrk2.1(ars11)cw+nit+*	Chlamydomonas Resource Center	(Moseley et al. 2009)
CC4270	*snrk2.2-1*	*snrk2.2(are10)cw+nit+mt+*	Chlamydomonas Resource Center	(Davies et al. 1999)
CC4273	*snrk2.2-2*	*snrk2.2(are16)cw+nit+*	Chlamydomonas Resource Center	(Moseley et al. 2009)
CC4281	*snrk2.1 snrk2.2-2*	*snrk2.1(ars11)snrk2.2(are16)*	Chlamydomonas Resource Center	(Moseley et al. 2009)
CC4276	*psr1 snrk2.1*	*psr1-1snrk2.1(ars11)cw+nit+*	Chlamydomonas Resource Center	(Moseley et al. 2009)
CC4272	*psr1 snrk2.2-1*	*psr1-1snrk2.2(are10)cw+nit+*	Chlamydomonas Resource Center	(Moseley et al. 2009)
CC4274	*psr1 snrk2.2-2*	*psr1-1snrk2.2(are16)cw+*	Chlamydomonas Resource Center	(Moseley et al. 2009)
CC4278	*psr1 snrk2.1 snrk2.2-1*	*psr1-1snrk2.1(ars11)snrk2.2(are10)cw+nit+*	Chlamydomonas Resource Center	(Moseley et al. 2009)

[a]Chlamydomonas Resource Center, USA strain ID number is used.
[b]References:

Davies, J. P., F. H. Yildiz, A. R. Grossman (1999). Sac3, an Snf1-like serine/threonine kinase that positively and negatively regulates the responses of Chlamydomonas to sulfur limitation. *Plant Cell* 11, 1179-1190

Moseley, J. L., D. Gonzalez-Ballester, W. Pootakham, S. Bailey, A. R. Grossman (2009). Genetic interactions between regulators of *Chlamydomonas* phosphorus and sulfur deprivation responses. *Genetics* 181, 889-905

Pröschold, T., E. H. Harris, A. W. Coleman (2005). Portrait of a species: *Chlamydomonas reinhardtii*. *Genetics* 170, 1601-1610

Shimogawara, K., D. D. Wykoff, H. Usuda, A. R. Grossman (1999). *Chlamydomonas reinhardtii* mutants abnormal in their responses to phosphorus deprivation. *Plant Physiology* 120, 685-693

Supplementary Table 2 Matched identifications for each significant lipid peak (in bold) based on PC1 and 2 loadings (Fig. 6b) and DF1 and 2 loadings (Supplemental Fig. 5b) derived from PCA and PC-DFA of UHPLC-MS analysis of wild type and mutant strains in response to P limitation. Lipid class definitions: GL02, diglycerides; GL03, triglycerides; GP00, phosphatidic acids; GP03, phosphatidylethanolamines; UN, unclassed lipids

idx	*m/z*	Lipid Class	PC1 Loading	PC2 Loading	DFA1 Loading	DFA2 Loading	Putatively assigned lipid identifications by accurate mass match with databases: HMDB, METLIN or KEGG
**1256**	599.41	GL02	0.015	**0.109**	-0.001	0.023	Diglycerols
**1412**	669.45	GP00	0.032	**0.112**	0.000	0.006	Glycerophosphates
**1608**	734.59	GL02	**0.580**	**0.178**	**0.197**	**-0.147**	DGTS (16:0/16:0)
**1611**	735.59	GL02	**0.276**	0.093	0.093	-0.071	DGTS (16:0/16:0)
**1615**	737.61	UN	**0.119**	-0.032	0.052	0.013	Cyclic archaeol with two cyclopentane rings or diglycerols
**1678**	756.57	GL02	**0.183**	**0.213**	0.086	**0.103**	DGTS (16:0/16:0)
**1680**	757.58	GL02	0.082	**0.103**	0.042	0.063	DGTS (16:0/16:0)
**1682**	758.59	UN	**0.344**	**-0.141**	0.072	**-0.316**	1-Octadecanoyl-2-(9Z,1Z-octadecadienoyl)-3-O-[(N,N,N-trimethyl) homoserine]-glycerol or DGTS (16:0/18:2(9Z,12Z))
**1684**	759.59	UN	**0.178**	-0.075	0.044	-0.139	1-Octadecanoyl-2-(9Z,1Z-octadecadienoyl)-3-O-[(N,N,N-trimethyl) homoserine]-glycerol or DGTS (16:0/18:2(9Z,12Z))
**1687**	761.61	GP00	**0.114**	0.053	0.032	-0.029	Glycerophosphates
**1717**	769.48	UN	**0.143**	0.090	0.024	-0.060	monoglycerols or diglycerols
**1756**	776.54	GP03	-0.018	**0.105**	0.009	0.098	Glycerophosphoserines
**2025**	845.66	UN	**0.107**	-0.096	0.038	-0.068	beta-hydroarchaetidylglyerol; diglycerol or triglycerol
**2035**	847.68	GL02	**0.178**	**-0.123**	0.071	-0.051	Diglycerols or possible triglycerols
**2074**	855.74	UN	-0.005	0.029	-0.035	**-0.135**	Galactosylceramide, Glucosylceramide or short chained triglycerols
**2193**	871.68	GL03	**0.176**	-0.039	0.046	**-0.117**	Triglycerols
**2199**	872.68	GL03	**0.105**	-0.024	0.032	-0.048	Triglycerols
**2208**	873.69	GL02	**0.150**	-0.039	0.054	-0.042	DGTS (24:1/24:0) or possible triglycerol
**2220**	875.71	GL03	**0.306**	**-0.142**	0.095	**-0.167**	Triglycerols
**2224**	876.71	GL03	**0.184**	-0.085	0.057	**-0.100**	Triglycerols
**2244**	879.74	GL03	-0.013	0.059	-0.055	**-0.190**	Triglycerols
**2252**	880.74	GL03	-0.005	0.035	-0.031	**-0.111**	Triglycerols
**2259**	881.76	GL03	-0.004	0.033	-0.066	**-0.272**	Triglycerols
**2264**	882.76	GL03	-0.003	0.020	-0.039	**-0.162**	Triglycerols
**2298**	887.57	UN	-0.004	**0.639**	-0.017	**0.198**	Glycerophosphoinositols; alpha,alpha'-Trehalose 6-mycolate or unclassified lipids
**2301**	888.57	UN	-0.001	**0.383**	-0.010	**0.116**	Glycerophosphoinositols; alpha,alpha'-Trehalose 6-mycolate or unclassified lipids
**2306**	889.57	UN	0.001	**0.122**	-0.001	0.045	Glycerophosphoinositols; alpha,alpha'-Trehalose 6-mycolate or unclassified lipids
**2393**	901.72	GL03	**0.124**	-0.068	0.057	-0.014	Triglycerols
**2671**	953.54	UN	0.027	**0.221**	-0.004	0.044	Glycerophosphoinositols or unclassified lipids
**2676**	954.54	UN	0.017	**0.137**	-0.002	0.026	Glycerophosphoinositols or unclassified lipids
